# An abrupt bleeding of the anteriorly- displaced sigmoid sinus: a rare complication of myringoplasty

**DOI:** 10.1186/s12901-017-0045-9

**Published:** 2017-12-06

**Authors:** Sarah Zaher Addeen, Mohammad Al- Mohammad

**Affiliations:** 1 Faculty of Medicine, Damascus University, Damascus, Syria; 2 Otorhinolaryngology Department, Al- Mouwassat University Hospital, Damascus, Syria

**Keywords:** Sigmoid sinus, Myringoplasty, Abnormally displaced, Anterior course, Anatomical variation

## Abstract

**Background:**

The location of the sigmoid sinus within the mastoid cavity is quite variable. An anteriorly- displaced vertical segment of the sigmoid sinus constitutes an uncommon but dangerous anatomical variation that surgeons rarely encounter during surgery. In this variation, the sigmoid sinus lays underneath a very thin bony flap, which makes it easily damaged. Thus, an abrupt fatal bleeding might occur. Despite the many hypotheses about its origin (Chronic otitis media, hypopneumatization, etc.), the pathogenesis of this variation is still not completely understood.

**Case presentation:**

We present a case where the vertical segment of the left sigmoid sinus was encountered just underneath a one- millimeter bony flap in the posterior wall of the external auditory canal during an attempted myringoplasty.

**Conclusion:**

Anatomical variations of the sigmoid sinus are not uncommon, and the otolaryngologist should be aware of such variations to prevent unpleasant, intra- operative surprises.

## Background

The location of the sigmoid sinus within the mastoid cavity is quite variable, and thus impacts surgical planning and execution profoundly [1]. An anteriorly- displaced vertical segment of the sigmoid sinus is an important anatomical variation that triggered a debate about its causality [1–4]. Some studies showed that the distance between the sigmoid sinus and the posterior wall of the external auditory canal is significantly smaller in patients with sclerotic mastoids due to chronic otitis media (COM) in childhood, or genetic factors that provoke mastoid hypopneumatization [2], while other studies refuted this claim [3], and hypothesized that volume reduction may result from the sclerotic change in the air cell system, rather than from shrinkage of the mastoid bone [5], and even suggested that the sinus location is responsible for decreasing the mastoid pneumatization [6]. This variation has been scarcely reported during surgery nevertheless, it might easily provoke a massive bleeding if the surgeon didn’t take caution to the abnormal sinus that lies underneath a millimeter- thin bony flap. [7–9]. The anteriorly- displaced sinus is a well-defined anomaly in high- resolution computed tomography imaging (HRCT) [10]. We report a case where the sigmoid sinus was encountered just underneath a one- millimeter bony flap in the posterior wall of the external auditory canal during an attempted myringoplasty.

## Case presentation

A 40- year- old female patient presented to Al Mouwassat Universityhospital outpatients’ clinics with hearing loss, and tinnitus in her left ear.

**Fig. 1:**
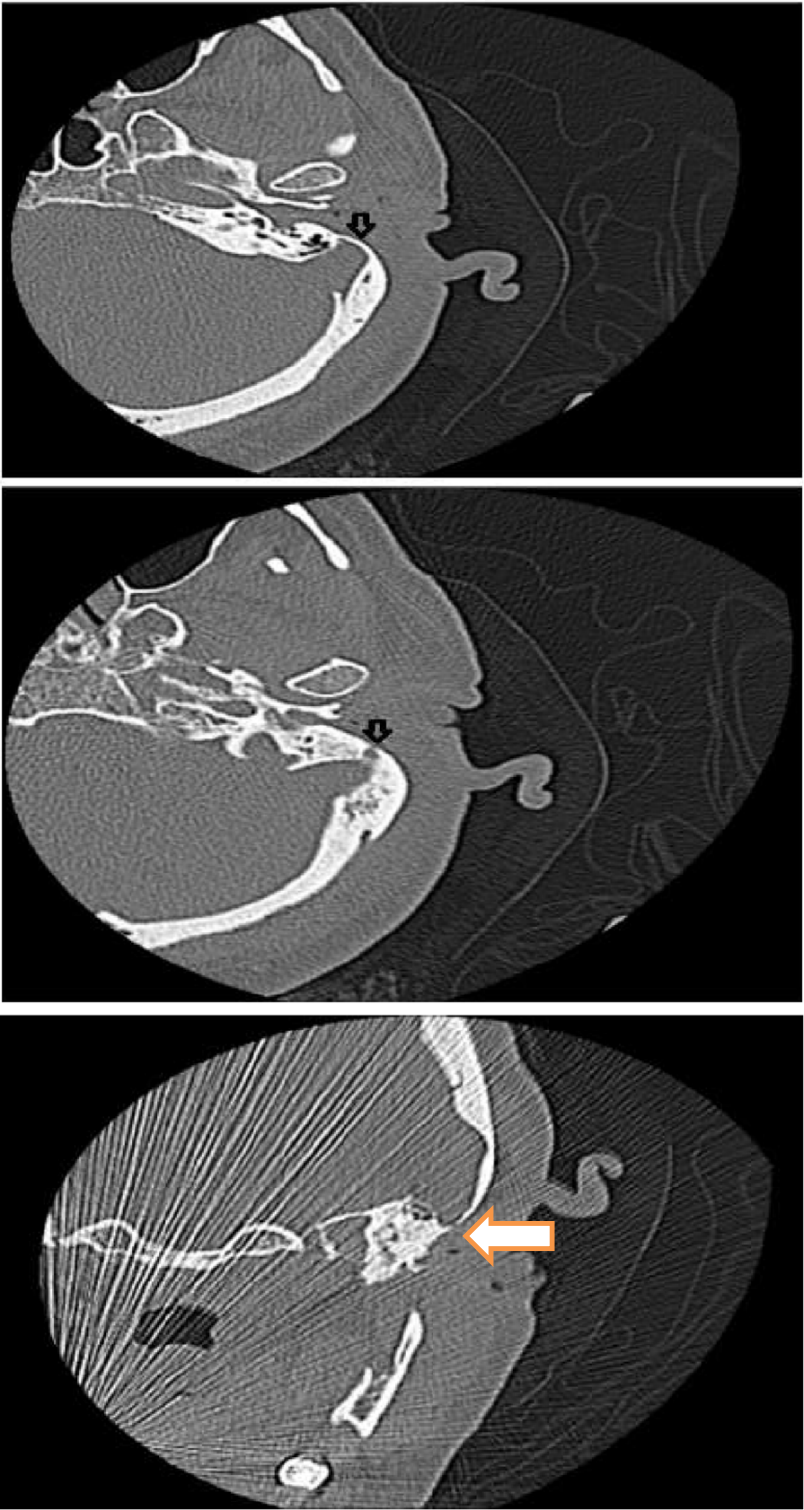
A postoperative high-resolution computed tomography (HRCT) of the temporal bone shows that the vertical segment of the left sigmoid sinus (arrow) is abnormally forward- displaced

**Fig. 2:**
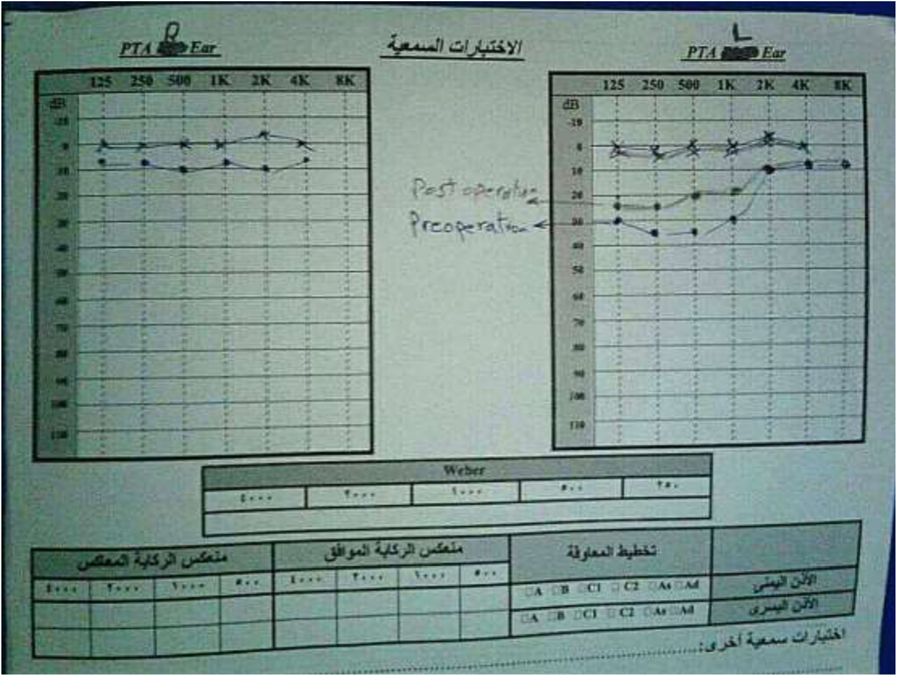
‌Pre and postoperative PTA (pure tone audiogram) of the patient that shows the decrease of air - bone gap to 22 db

She had a history of acoustic trauma 3 years earlier. On physical examination, the external ear canal was normal. A sclerotic –edged perforation was found to involve the whole antero- inferior quadrant of the tympanic membrane. Her thorough otorhinolaryngolgical examination was otherwise normal. The pure tone audiogram (PTA) demonstrated a conductive hearing loss with 32 dB gap in the left ear; hearing on the right side was within normal. Left myringoplasty was scheduled. Entry was via the post- auricular approach. Then the temporalis fascia superficialis was harvested. After that, the triangular musculoperiosteal flap was anteriorly elevated to the level of the meatus.A sudden venous profuse bleeding from the posterior wall of the external bony canal was encountered as soon as the tympanosquamous suture was reached. It seemed like there is a millimeter- thin bony shell covering this wall that fractured with a slight touch from the knife. The bleeding was controlled by gauze followed by gelfoam packing successfully, and the operation was completed thereafter. The middle ear was inspected, the ossicles were intact and mobile. No abnormal vascular structure was seen. The packing was removed at the end of the operation. The patient received antibiotic treatment with Ceftriaxone 500 mg twice daily for 10 days. A postoperative high-resolution computed tomography (HRCT) of the temporal bone showed that the vertical segment of the left sigmoid sinus was abnormally anteriorly- displaced; dehiscent to the posterior wall of the external auditory canal (Fig. 1). The posterior wall of the lateral part of the bony external auditory meatus was defective and thinned by the sigmoid sinus that lays just underneath a 1- mm flap of bone. A PTA was obtained 2 months after the surgery, and revealed that the conductive hearing loss gap improved to 22 dB (Fig. 2).

## Discussion

The Sigmoid sinus originates at the junction of the transverse and the superior petrosal sinuses at the superior border of the petrous bone, from this point on goes medially in the vertical plane toward the medial portion of the mastoid cavity, sculpturing a deep, S- shaped canal and terminates anteriorly at the jugular bulb that descends from the jugular foramen as a jugular vein [11].

An anterior course of the sigmoid sinus is a rare anatomical variation that has been reported in the medical literature. Gangopadhyay et al. reported a similar case to ours, as they came upon the sigmoid sinus underneath the skin of the posterior wall of the external auditory canal during an attempted myringoplasty [7], while Ulug and colleagues confronted massive bleeding from an anteriorly- displaced sigmoid sinus during stapedectomy [9].Moreover, Puraviappan and colleagues discovered the abnormal anterior course of a ruptured sigmoid sinus in a referral case of middle ear and mastoid exploration via post- auricular approach [8]. There has been a controversy on whether the abnormally- located sigmoid sinus in the mastoid cavity plays a role in otologic pathologies, or the variation itself is a consequence of pathology. Some studies showed that the distance between the sigmoid sinus and the posterior wall of the external auditory canal is significantly smaller in patients with sclerotic mastoids due to chronic otitis media (COM) in childhood, or genetic factors that provoke mastoid hypopneumatization [2], while other studies refuted this claim [3], and hypothesized that volume reduction may result from the sclerotic change in the air cell system, rather than from shrinkage of the mastoid bone [5], and even suggested that the sinus location is responsible for decreasing the mastoid pneumatization [6]. From an embryologic aspect, The degree of pneumatization of the mastoid varies greatly in normal temporal bones [12]. The age at which the gas cells develop is subject to huge individual variation, as well as the number, size and volume of the mastoidian cells which are considered as individual characteristics. In fact, many factors impact the growth and pneumatization of the mastoid, especially heredity, environment, nutrition, gas exchanges and frequency of infections. In addition, anatomic variability of adjacent structures affect the development of the mastoid pneumatization and vice versa [13].

An antero- medially displaced sinus has been described in patients with Ménière’s disease, the researchers justified this finding as a result of tightened Trautmann’s triangle in these patients [4]. Nevertheless, it’s not infrequent to catch this same variation in disease- free temporal bone as stated by Sarmiento and colleagues [1], Ulug and colleagues [9], and as we suggest in our case.

In our patient, we stopped the sinusoidal bleeding by gauze and gelfoam packing. This method was applied by other authors to control anomalous jugular bulb hemorrhage during middle ear surgery [14]. This method, however, implicates the risk of intracranial venous hypertension, especially when the dominant sinus is occluded (usually the right sinus), the sinus was previously healthy, and no patent connection exists between the two sigmoid sinuses via transverse sinuses [15]. Yet, we didn’t face this complication in our patient, regarding that we packed the left -non dominant- sinus and that the two sigmoid sinuses were normally connected. After 2 months of follow- up, the patient is still doing well, and no evidence of intracranial hypertension showed up. In this case, we used the temporalis fascia to close the tympanic membrane perforation. The temporalis fascia is widely used in myringoplasty [16]. Some studies suggest that the hearing results of the temporalis fascia graft are comparable to those of the cartilage palisades, especially in large perforations [16, 17]. In contrary, other studies suggest that the anatomical success rate for a cartilage myringoplasty is higher than for a fascia one, with no significant difference on the functional results [18].

Although high-resolution computed tomography imaging (HRCT) was not performed to our patient preoperatively since it’s not routinely performed in our hospital, it is still a crucial investigation in temporal bone disorders management and anatomical variations detection [10]. It’s an accurate tool to evaluate mastoid pneumatization and aditus ad antrum blockage (which play a role in failure of myringoplasty) [19].

However, this imaging technique has its limitations in regard to detecting the dehiscence of some anatomic structures such as facial canal, lateral semicircular canal, and tegmen [20]. In addition, we noticed that there is a scarce in studies that investigate HRCT importance in first attempt myringoplasty whereas most of them focused on revision myringoplasty.

A study compared the intraoperative data and computed tomography measures of 30 patients and concluded that the a tomographic distance between the sigmoid sinus and the external ear canal measuring less than 9 mm complicated the procedure, their lowest distance was 4.7 mm [21].

Another study measured the same distance as 13.2 mm [22]. In this report, the distance was about 1 mm, which exposed the sinus to hemorrhage jeopardy.

## Conclusion

Anatomical variations of the sigmoid sinus are not uncommon. The otolaryngologist should be aware of such variations to prevent unpleasant, intra- operative surprises. In addition, if HRCT was performed before surgery for any reason, the sigmoid sinus should be carefully inspected.

## Abbreviations

COM: Chronic otitis media
dB: Decibles
HRCT: High- resolution computed tomography imaging
PTA: Pure tone audiogram

## References

[1] Sarmiento PB, Eslait FG (2004). Surgical classification of variations in the anatomy of the sigmoid sinus. Otolaryngol Head Neck Surg.

[2] Shatz A, Sade J (1990). Correlation between mastoid pneumatization and position of the lateral sinus. Ann Otol Rhinol laryngol.

[3] Orr JB, Wendell Todd N (1988). Jugular bulb position and shape are unrelated to temporal bone pneumatization. Laryngoscope.

[4] Paparella M, Sajjadi H: The lateral sinus and Trautmann’s triangle in Meniere’s disease: considerations of pathogenesis. In: Proceedings of the Second International Symposium on Menieres’s Disease Amsterdam: Kugler Publications: 1988; 1988.

[5] Lee D-H, Jung M-K, Yoo Y-H, Seo J-H (2008). Analysis of unilateral sclerotic temporal bone: how does the sclerosis change the mastoid pneumatization morphologically in the temporal bone?. Surg Radiol Anat.

[6] Çam OH, Karatas M (2015). A life threatening pitfall in ear surgery: extracranial sigmoid sinus. J Craniofac Surg.

[7] Gangopadhyay K, McArthur P, Larsson S (1996). Unusual anterior course of the sigmoid sinus: report of a case and review of the literature. J Laryngol Otol.

[8] Puraviappan P, Prepageran N, Ong CA, Abd KR, Lingham OR, Raman R (2014). An abnormal sigmoid sinus with a dire clinical implication. Ear Nose Throat J.

[9] Ulug T, Basaran B, Minareci O, Aydin K (2004). An unusual complication of stapes surgery: profuse bleeding from the anteriorly located sigmoid sinus. Eur Arch Otorhinolaryngol.

[10] Visvanathan V (2015). Anatomical variations of the temporal bone on high-resolution computed tomography imaging: how common are they?. J Laryngol Otol.

[11] Cummings CW, et al. Otolaryngology Head & Neck Surgery, Vol-5. Elsevier; 1998:3700.

[12] Ars B, Dirckx J, Ars-Piret N, Buytaert J (2012). Insights in the physiology of the human mastoid: message to the surgeon. Int Adv Otol.

[13] Ars B, Ars-Piret N (1997). Morpho-functional partition of the middle ear cleft. Acta Otorhinolaryngol Belg.

[14] Moore PJ (1994). The high jugular bulb in ear surgery: three case reports and a review of the literature. J Laryngol Otol.

[15] Sekhar LN, Tzortzidis FN, Bejjani GK, Schessel DA (1997). Saphenous vein graft bypass of the sigmoid sinus and jugular bulb during the removal of glomus jugulare tumors: report of two cases. J Neurosurg.

[16] Kalcioglu M, Tan M, Croo A (2013). Comparison between cartilage and fascia grafts in type 1 tympanoplasty. B-ENT.

[17] Arun K, Shakya D (2014). Comparison of outcomes of Myringoplasty with cartilage palisades and Temporalis fascia in large perforations. Otolaryngol Head Neck Surg.

[18] Yegin Y, Çelik M, Koç AK, Küfeciler L, Elbistanlı MS, Kayhan FT (2016). Comparison of temporalis fascia muscle and full-thickness cartilage grafts in type 1 pediatric tympanoplasties. Braz J Otorhinolaryngol.

[19] El-kady AS, Haroun Y, Kassem KM, Galal O (2009). The value of computed tomography scanning in assessment of Aditus ad Antrum patency and choice of treatment line in revision Myringoplasty. Med J Cairo Univ.

[20] Tatlipinar A, Tuncel A, Öğredik EA, Gökçeer T, Uslu C (2012). The role of computed tomography scanning in chronic otitis media. Eur Arch Otorhinolaryngol.

[21] Pereira AR, Pinheiro SD, de Castro JDV, Ximenes Filho JA, de Freitas MR (2012). Mastoidectomy: anatomical parameters x surgical difficulty. Arquivos Internacionais de Otorrinolaringologia.

[22] Fkinci G, Ko A, Baltaciǧlu F, Veyseller B, Altintaş O, Han T. Temporal bone measurements on high-resolution computed tomography. J Otolaryngol. 2004;33(6):33–6. 10.2310/7070.2004.03018 15971656

